# Health-Related Quality of Life in European Childhood Cancer Survivors: Protocol for a Study Within PanCareLIFE

**DOI:** 10.2196/21851

**Published:** 2021-01-25

**Authors:** Gabriele Calaminus, Katja Baust, Claire Berger, Julianne Byrne, Harald Binder, Leonie Casagranda, Desiree Grabow, Martha Grootenhuis, Peter Kaatsch, Melanie Kaiser, Tomas Kepak, Kateřina Kepáková, Leontien C M Kremer, Jarmila Kruseova, Ales Luks, Claudia Spix, Marleen van den Berg, Marry M M van den Heuvel-Eibrink, Eline van Dulmen-den Broeder, Rahel Kuonen, Grit Sommer, Claudia Kuehni

**Affiliations:** 1 Department of Paediatric Haematology and Oncology University Hospital Bonn Bonn Germany; 2 Department of Paediatric Haematology and Oncology University Hospital Münster Münster Germany; 3 Department of Paediatric Hematology and Oncology Unit University Hospital of Saint-Étienne Saint-Étienne France; 4 Boyne Research Institute Drogheda Ireland; 5 Institute of Medical Biometry and Statistics Faculty of Medicine and Medical Center University of Freiburg Freiburg Germany; 6 Host Research Team EA4607 SNA-EPIS (Autonomic Nervous System, Epidemiology, Physiology, Exercise, and Health) Jean Monnet University of Saint-Etienne PRES (Education and Research Cluster) Lyon St. Etienne France; 7 Division of Childhood Cancer Epidemiology, German Childhood Cancer Registry Institute of Medical Biostatistics, Epidemiology and Informatics (IMBEI) University Medical Center of the Johannes Gutenberg University Mainz Mainz Germany; 8 Princess Máxima Center for Pediatric Oncology Utrecht Netherlands; 9 University Hospital Brno Masaryk University Brno Czech Republic; 10 International Clinical Research Center (FNUSA-ICRC) Masaryk University Brno Czech Republic; 11 DCOG LATER Utrecht Netherlands; 12 Department of Paediatric Haematology/Oncology Second Faculty of Medicine Charles University Prague Czech Republic; 13 Department of Paediatric Oncology Emma Children’s Hospital Amsterdam UMC, Vrije Universiteit Amsterdam Amsterdam Netherlands; 14 Sophias Childrens Hospital Erasmus MC Rotterdam Netherlands; 15 Swiss Childhood Cancer Registry Institute of Social and Preventive Medicine University of Bern Bern Switzerland; 16 Pediatric Endocrinology, Diabetology and Metabolism Department of Pediatrics Inselspital, Bern University Hospital Bern Switzerland; 17 Pediatric Oncology Inselspital, Bern University Hospital University of Bern Bern Switzerland

**Keywords:** children, adolescents, neoplasms, quality of life, health status, Europe, epidemiology, survivors of childhood cancer

## Abstract

**Background:**

Survival after childhood cancer has improved to more than 80% during the last few years, leading to an increased number of childhood cancer survivors. Cancer itself, or its treatment, may cause chronic health conditions, including somatic and mental sequelae, which may affect survivors’ health-related quality of life (HRQoL).

**Objective:**

The project PanCareLIFE aims to establish a large database with comprehensive data on childhood cancer survivors from different European countries, including data on HRQoL. Within PanCareLIFE, this study aims to describe HRQoL in survivors, investigate predictors of HRQoL, and describe the association of HRQoL with hearing and female fertility impairment. This paper describes the design of the HRQoL study, the origin of data, strategies for data collection, and sampling characteristics of survivors from each contributing country.

**Methods:**

A total of 6 institutions from 5 European countries (the Czech Republic, France, Germany, the Netherlands, and Switzerland) provided data on HRQoL assessed with the Short Form 36 and on relevant predictors. The central PanCareLIFE data center aggregated the data and harmonized the variables between the institutions. Survivors were eligible if they received a diagnosis of cancer according to the 12 main groups of the International Classification of Childhood Cancer, 3rd edition, or Langerhans cell histiocytosis; were aged ≤18 years at the time of diagnosis; were residents of the respective country at the time of diagnosis; had survived ≥5 years after cancer diagnosis; were aged ≥18 years at the time of the questionnaire survey; and did not refuse to registration in the national or local childhood cancer cohort.

**Results:**

We identified 24,993 eligible survivors. Of those, 19,268 survivors received a questionnaire and 9871 survivors participated, resulting in response rates of 9871/24,993 (39.50%) of eligible survivors and of 9871/19,268 (51.23%) invited survivors. Most participants were diagnosed with cancer between the ages of 10 and 14 years (3448/9871, 34.93%) or <5 years (3201/9871, 32.43%). The median age was 8 years. Of the 9871 participants, 3157 (31.97%) were survivors of leukemia, 2075 (21.02%) lymphoma, and 1356 (13.7%) central nervous system (CNS) tumors. Most participants (9225/9871, 93.46%) had no history of a subsequent tumor; 77.45% (7645/9871) received chemotherapy with or without other treatments. More than half (5460/9871, 55.31%) were aged 25 to 34 years at the time of the HRQoL study. Participating survivors differed from nonparticipants; participants were more often women, survivors of leukemia or lymphoma, and less frequently, survivors of CNS tumors than nonparticipants.

**Conclusions:**

PanCareLIFE successfully assessed HRQoL and its predictors in 9871 European survivors of childhood cancer. This large population will permit detailed investigations of HRQoL after childhood cancer, particularly the impact of hearing and female fertility impairment on HRQoL.

**International Registered Report Identifier (IRRID):**

RR1-10.2196/21851

## Introduction

At present, more than 80% of children diagnosed as having cancer in Europe survive [1]. Almost half a million childhood cancer survivors are estimated to live in Europe in 2020 [2]. However, cancer itself, or its treatment, causes chronic health conditions, including a broad spectrum of somatic [3] and mental sequelae [4]. In the St. Jude Lifetime Cohort Study, nearly all childhood cancer survivors had at least one chronic health condition by the age of 50 years and twice the burden of disease than the general population [5]. Chronic health conditions such as heart failure, second neoplasms, or pulmonary dysfunction can be life threatening; other health conditions such as fertility and hearing impairment can affect survivors’ life planning and daily life, which may reduce their health-related quality of life (HRQoL) [6-8].

HRQoL is a multidimensional concept that includes elements of physical, functional, social, and psychological health as well as perceived health status and well-being [9]. Many studies assessing HRQoL in childhood cancer survivors used different questionnaires or different reference groups or varied in characteristics of the study population (eg, treatment era, age of survivors, cancer diagnostic groups), making it difficult to compare results between studies [10]. Results from large childhood cancer survivor studies in the United Kingdom (British Childhood Cancer Survivor Study, BCCSS) [11], the United States (Childhood Cancer Survivor Study) [12], and Switzerland (Switzerland Childhood Cancer Survivor Study, SCCSS) [6] suggest that, on average, childhood cancer survivors have similar HRQoL compared with the general population. However, there were significant differences in HRQoL between subgroups of survivors. Women, survivors with low educational background, survivors of brain tumors, and survivors who had undergone radiotherapy had the lowest HRQoL [6,11,12]. It is still unclear which other factors influence HRQoL and whether HRQoL in childhood cancer survivors differs among European countries.

Within the PanCareLIFE project, funded by the European 7th Framework Program (FP7), we aim to study HRQoL in a large database of childhood cancer survivors from 5 European countries using a homogeneous approach to assess and analyze HRQoL. In particular, we aimed to compare HRQoL in European childhood cancer survivors with normative data and between European countries to determine predictors of HRQoL and describe the effect of hearing and fertility impairment on HRQoL [13]. This study provides an overview of the design, data origin, and data collection strategies and summarizes the characteristics of survivors who participated in this study.

## Methods

### The PanCareLIFE Research Framework

The European FP7 project PanCareLIFE (grant agreement no. 602030) started in 2013 [14]. Institutions from 10 countries provided data on more than 15,000 childhood, adolescent, or young adult cancer survivors. Within the PanCareLIFE framework, this study focused on long-term HRQoL in childhood cancer survivors [13]. It was based in the University Hospitals of Münster (2013-2015) and Bonn (2016-2018). The Institute of Social and Preventive Medicine at the University of Bern provided methodological support and conducted the analyses. Each institution obtained ethical approval according to their local and/or national authority regulations before collecting the data. For all survivors, either written informed consent was obtained or the ethics committee agreed that an individual’s consent was not required for this questionnaire study.

### Origin of Data and Inclusion Criteria

The PanCareLIFE HRQoL study population was composed of 6 national or regional cohorts, which had slightly different inclusion criteria. Table 1 provides an overview of the countries and institutions that provided data for the PanCareLIFE HRQoL study.

**Table 1:** Sources of eligible survivors for the PanCareLIFE health-related quality of life study.

Country	Data provider	National or regional cohort	Source of baseline data	Identification (prospective or retrospective)	Estimated national or regional coverage (%)
Switzerland	UNIBE^a^	SCCSS^b^ [15]	National population–based registry	Prospective	95 [16]
The Czech Republic	FNM^c^ and UHB^d^	N/A^e^	Hospital-based cohort	Retrospective	95
France	CHU-SE^f^	Rhone-Alpes Cohort	Regional population–based registry	Prospective and retrospective	95
The Netherlands	DCOG LATER^g^ Registry with data from 7 pediatric oncology hospitals	DCOG LATER [17]	Registry based on nationwide hospital cohorts [18]	Retrospective	>95
Germany	VIVE^h^ group (UKB^i^/UKM^j^)	VIVE	National population–based registry [19]	Prospective	>95 [20]

^a^UNIBE: University of Bern.

^b^SCCSS: Swiss Childhood Cancer Survivor Study.

^c^FNM: Motol Teaching Hospital, Prague, the Czech Republic.

^d^UHB: University Hospital Brno, the Czech Republic.

^e^N/A: not applicable.

^f^CHU-SE: Centre Hospitalier Universitaire de Saint-Étienne, St Étienne, France.

^g^DCOG LATER: Dutch Childhood Oncology Group Survivor study.

^h^VIVE: First Basic Survey on Life Situation, State of Health, and Quality of Life of Childhood Cancer Survivors in Germany.

^i^UKB: Universitätsklinikum, Bonn, Germany.

^j^UKM: Universitätsklinikum, Münster, Germany.

We combined data from Brno and Prague to 1 Czech cohort. The identification of eligible survivors depended on the respective cohort. Switzerland and Germany prospectively identified the survivors from the national childhood cancer registry, the Czech Republic and the Netherlands retrospectively identified the survivors and France implemented both ways of identification (Table 1) [15,17-19]. The identification of survivors was population-based in Switzerland, the Rhone-Alpes region (France), and Germany. In the Netherlands and the Czech Republic, it was hospital-based, with an estimated coverage of 95% of the national childhood cancer population. Switzerland, Germany, and the Netherlands enrolled their cohorts before and France and the Czech Republic after the launch of PanCareLIFE. Details on the period of data collection and sample characteristics of each cohort are listed in Table 2.

**Table 2:** Number of eligible patients, time period of cancer diagnosis, age of survivors at diagnosis, time period of the health-related quality of life (HRQoL) survey, and age of survivors at HRQoL survey, by country.

Country	Number of eligible survivors	Years of diagnosis	Age at diagnosis (years)	Years of study	Age at study (years)
Switzerland	3023	1976-2010	≤18	2007-2016	18-47
The Czech Republic	3127	1967-2010	≤18	2013-2017	18-51
France	1060	1987-1999	≤15	2005-2016	18-43
The Netherlands	5639	1963-2001	≤17	2016-2017	18-70
Germany	12,144	1980-2003	≤15	2014-2015	25-47

Survivors were eligible for the study if they were aged ≤18 years at the time of diagnosis, had been residents of the respective country at the time of diagnosis, had a cancer diagnosis according to the International Classification of Childhood Cancer, 3rd edition (ICCC-3) [21] or had been diagnosed as having Langerhans cell histiocytosis, had survived ≥5 years after cancer diagnosis, were aged ≥18 years at the time of the questionnaire survey, were not undergoing treatment for cancer at the time of the study, and did not refuse to registration in the national or local childhood cancer cohort. The last criterion did not apply to Czech survivors because patients were identified in a clinical routine. We could not calculate the exact age at the time of survey and the survival time, as we did not receive information on the day of birth, day of diagnosis, and day of survey from the institutions because of PanCareLIFE data protection rules. Therefore, we allowed for a tolerance of 2 months and included survivors who were 2 months older than 18 years at the time of cancer diagnosis, those who survived 2 months less than 5 years, and those who were 2 months younger than 18 years at the time of the survey.

In addition to the general eligibility criteria, there were country-specific reasons for the exclusion of eligible survivors and not all reasons applied to all cohorts. The Czech Republic, France, and the Netherlands excluded survivors with severe mental sequelae. In Switzerland and the Czech Republic, survivors were excluded from invitation because of physicians’ decisions mainly because of palliative care or relapsed disease. France, the Czech Republic, and Switzerland did not exclude survivors because of competing surveys. In the Netherlands and Germany, 2.21% (552/24,993) of survivors could not be included because they were already enrolled in competing surveys (Table 3). The French cohort did not include leukemia survivors.

**Table 3:** Overview of eligible, invited, and participating survivors in the health-related quality of life Short Form 36 questionnaire study, overall and by country.

Overview		Country					
		All data providers combined (n=24,993), n (%)	Switzerland (n=3023), n (%)	The Czech Republic (n=3127), n (%)	France (n=1060), n (%)	The Netherlands (n=5639), n (%)	Germany (n=12,144), n (%)
**Eligible cohort**							
	Died before questionnaire mailing	2088 (8.35)^a^	252 (8.34)^a^	6 (0.19)^a^	74 (6.98)^a^	677 (12.00)^a^	1079 (8.89)^a^
	Severe mental sequelae	28 (0.11)^a^	0 (0)^a^	15 (0.48)^a^	2 (0.19)^a^	11 (0.20)^a^	0 (0)^a^
	Living abroad	489 (1.96)^a^	95 (3.14)^a^	8 (0.26)^a^	0 (0)^a^	127 (2.25)^a^	259 (2.13)^a^
	No address or lost-to-follow-up	854 (3.42)^a^	148 (4.90)^a^	259 (8.28)^a^	122 (11.51)^a^	16 (0.28)^a^	309 (2.54)^a^
	Physician’s decision not to invite the survivor^b^	118 (0.47)^a^	14 (0.46)^a^	104 (3.33)^a^	0 (0)^a^	0 (0)^a^	0 (0)^a^
	Competing surveys	552 (2.21)^a^	0 (0)^a^	0 (0)^a^	0 (0)^a^	266 (4.72)^a^	286 (2.36)^a^
	Other^c^	1596 (6.39)^a^	1 (0.03)^a^	1159 (37.06)^a^	11 (1.04)^a^	292 (5.18)^a^	133 (1.08)^a^
**Invited cohort**		19,268 (77.09)^a^	2513 (83.13)^a^	1576 (50.40)^a^	851 (80.28)^a^	4250 (75.37)^a^	10,078 (82.99)^a^
	Did not respond to the invitation	8277 (42.96)^d^	775 (30.84)^d^	461 (29.25)^d^	418 (49.12)^d^	2074 (48.80)^d,e^	4549 (45.14)^d^
	Refused to participate	914 (4.74)^d^	148 (5.89)^d^	30 (1.90)^d^	42 (4.94)^d^	0 (0)^d,e^	694 (6.89)^d^
	Short Form 36 information incomplete for full scoring	206 (1.07)^d^	5 (0.20)^d^	56 (3.55)^d^	6 (0.71)^d^	30 (0.71)^d^	109 (1.08)^d^
Participating cohort		9871 (51.23)^d^	1585 (63.07)^d^	1029 (65.29)^d^	385 (45.24)^d^	2146 (50.49)^d^	4726 (46.89)^d^

^a^Proportions of survivors from the eligible cohort.

^b^Physician’s decision not to invite the survivors because of psychosocial reasons or family problems.

^c^Other reasons were as follows: patients could not be approached during the study period (the Czech Republic, n=1156), ethical approval not obtained in time (the Netherlands, n=279), moved to another center (the Netherlands, n=3), unknown case at the time of the study (France, n=1), transgender (the Netherlands, n=1), did not understand the local language (Switzerland, n=1), unknown reasons (the Czech Republic, n=3; the Netherlands, n=9; France, n=11; Germany, n=133).

^d^Proportions of survivors from the invited cohort.

^e^Dutch survivors who refused to participate were included in the category *Did not respond to the invitation* because the Netherlands did not distinguish between nonresponse and refusal.

### Variables

Exposure variables and confounders covered the following topics: demographic characteristics, socioeconomic measures, lifestyle, cancer diagnosis, cancer treatment, hearing impairment, and female fertility impairment.

The outcome variables were the 36 HRQoL questions from the Short Form 36 (SF-36, version 1 or 2). The Netherlands, Germany, and the Czech Republic used version 1 of the SF-36, and Switzerland and France used version 2 [22,23]. Both versions differ only slightly in a few items, whereas the psychometric properties are comparable [24]. With the exception of variables concerning demographic characteristics, cancer diagnosis, and cancer treatment, all variables were self-reported.

Self-reported confounder variables included living with a partner (yes or no), education, occupational status, migration background, alcohol consumption, smoking, and body mass index. Another substudy of the PanCareLIFE research project investigated female fertility impairment and coded the data using 8 different criteria into a binary variable (fertility impairment: yes or no). The detailed procedure is outlined by van den Berg et al [25]. Fertility impairment data on male survivors were unfortunately not available in most of the cohorts; therefore, we will analyze female survivors only.

Hearing impairment data (yes or no) were collected together with information on HRQoL via questionnaires. Questions on hearing impairment differed slightly between data providers. Data on hearing impairment were not available for the Dutch cohort. All relevant variables (outcomes, exposures, and confounders) are listed in Multimedia Appendix 1.

The central PanCareLIFE data center defined a baseline variable list, including sex, cancer, and treatment-related variables and a minimal set of information provided for nonparticipants. The data center collected and harmonized the variables from each institution. We also recoded a few variables used specifically for the HRQoL study (eg, on hearing impairment) in cooperation with the institutions. All partners transferred the data according to the PanCareLIFE data protection standards.

The sources of cancer-related and treatment information, sources of self-reported data, and the methods of written questionnaire assessments differed slightly among the 5 cohorts (Table 4).

**Table 4:** Sources of cancer-related and treatment data and logistics of paper-pencil questionnaire assessment of health-related quality of life, by country.

Country	Source of cancer-related data	Source of treatment data	Logistics of questionnaire mailing to assess HRQoL^a^ and sociodemographic data
Switzerland	Forms sent by clinical sites to Swiss Childhood Cancer Registry	ITT^b^-based information complemented with AT^c^ information; AT data retrospectively collected from medical records	Coordinated centrally by SCCSS^d^. Survivors were approached by email and phone call reminders.
The Czech Republic	Clinical records in UHB^e^ and FNM^f^	AT information, retrospectively collected from medical records	Coordinated either by UHB or FNM. Survivors were approached during clinical visits, by email or phone calls, followed by mailed questionnaire.
France	Forms sent by clinical sites of the Rhône-Alpes region to the Rhône Alpes Regional Childhood Cancer Registry	AT information, retrospectively collected from medical records	Coordinated by CHU-SE^g^. Survivors were approached mainly by email and phone call reminders, sometimes during clinical visits, followed by mailed questionnaire.
The Netherlands	DCOG^h^ LATER Registry [17,18]	AT information, retrospectively collected from medical records	Coordinated by DCOG LATER^i^ clinics. Most survivors were approached by email and few during clinical visits.
Germany	Forms sent by clinical sites to the German Childhood Cancer Registry	ITT-based information	Coordinated centrally by UKM^j^ or UKB^k^ and GCCR^l^. Survivors were approached by mailed questionnaire.

^a^HRQol: health-related quality of life.

^b^ITT: intention to treat.

^c^AT: as treated.

^d^SCCSS: Swiss Childhood Cancer Survivor Study.

^e^UHB: University Hospital Brno, the Czech Republic.

^f^FNM: Motol Teaching Hospital, Prague, the Czech Republic.

^g^CHU-SE: Centre Hospitalier Universitaire de Saint-Étienne, Saint-Étienne, France.

^h^DCOG: Dutch Childhood Oncology Group.

^i^DCOG LATER: Dutch Childhood Oncology Group Survivor study.

^j^UKM: Universitätsklinikum, Münster, Germany.

^k^UKB: Universitätsklinikum, Bonn, Germany.

^l^GCCR: German Childhood Cancer Registry.

Data on HRQoL and HRQoL-specific exposure variables and self-reported confounders were assessed using the same questionnaire. An exception were the Dutch survivors, who answered the questionnaire on HRQoL on average 4 years later than the questions on sociodemographic characteristics and lifestyle for the purpose of their national Dutch Childhood Oncology Group Survivor (DCOG LATER) study.

### SF-36 Data From the General Population

Country-specific reference data from the general population did not exist for Switzerland, France, and the Czech Republic at the time of the study. We used the German SF-36 version 1 normative data from 1998 for norm-based scoring of data from all participating countries [26]. This norm-based scoring allowed us to compare the HRQoL between countries [24]. We also scored HRQoL raw data with more recent SF-36 version 2 normative data from Germany (2008-2011) [24] and from Switzerland (2015-2016) [27].

### Statistical Analyses

We compared survivors who participated in the survey with those who did not participate using chi-square tests. We used the software package Stata (version 14, StataCorp) for all analyses.

For all future analyses, we documented the planned statistical approaches for all HRQoL-related research questions of PanCareLIFE in a statistical analysis plan before we received the data from the central data center. In brief, SF-36 consists of 36 items (questions), which use a 2- to 6-point Likert scale, depending on the item. Between 2 and 10 items can be summarized into 1 of the 8 scales (physical functioning, role physical, bodily pain, general health, vitality, social functioning, role emotional, and mental health) and further into 2 summary scores (Physical Component Summary and Mental Component Summary). Because we will perform comparisons with both version 1 and version 2 normative data, we will transform all scales into the respective version with an algorithm provided by the developer of SF-36 [23,24,26]. We will convert raw scores into *T*-scores (mean 50, SD 10), and depending on the normative data used, we will stratify for subgroups, for example, age and sex. Higher scores indicate better HRQoL. Survivors with >50% missing data in any of the SF-36 scales will be excluded from the analyses. We will implement appropriate measures to deal with missing values, including imputation procedures. We will use regression analyses with HRQoL as an outcome variable to address all research questions to (1) compare HRQoL in European childhood cancer survivors with normative data and between European countries and to determine predictors of HRQoL, (2) describe the effect of hearing impairment on HRQoL, and (3) describe the effect of fertility impairment on HRQoL. We will include country, gender, and age in all multivariable models. We will also include the most important risk factors as defined by a selection criterion of P<.05 in the univariable regression. Multimedia Appendix 1 gives an overview of the variables that we plan to include in the regression analyses. If HRQoL scores have a skewed distribution, we will run logistic regressions (poor HRQoL vs normal or high HRQoL) and define poor HRQoL and high HRQoL, respectively, as scores below [11] and above the 10th percentile of the control population.

We will analyze subcohorts depending on the research question (Multimedia Appendix 2). For the analyses of differences in HRQoL between participating countries and predictors of HRQoL and for the analyses of the effect of hearing on HRQoL, we will use 2 main subsets for the analysis. In the first, we will include all survivors aged ≥25 years at the time of the study from all participating countries. The second subset will include survivors aged <25 years from all countries except Germany because Germany did not collect data in this age group. For the analyses on the effect of fertility impairment on HRQoL, we will investigate female survivors aged ≥25 years only. We will describe the detailed procedure in the respective publication of each research question.

## Results

We identified 24,993 eligible 5-year childhood cancer survivors (Table 3). Of those, 5725 had died before the questionnaire survey, had severe mental problems, were living abroad at the time of the study, had no available contact data (lost to follow-up), were enrolled in competing surveys, their physicians decided not to invite them, or for other reasons. Of the 19,268 survivors who received an invitation to the HRQoL survey, 9192 did not respond or refused to participate and 206 had incomplete SF-36 information, preventing full scoring. In total, 9871 survivors (4725 men and 5146 women) participated in the HRQoL analyses, thus resulting in response rates of 39.50% (9871/24,993) of eligible survivors and of 51.23% (9871/19,268) of invited survivors. Of the 9871 participating survivors, almost half were from Germany 4726 (47.88%), 2146 (21.74%) from the Netherlands, 1585 (16.06%) from Switzerland, 1029 (10.42%) from the Czech Republic, and 385 (3.90%) from France (Table 5).

**Table 5:** Sociodemographic and medical characteristics of participating survivors, by country.

Country		All data providers combined (N=9871), n (%)	Switzerland (n=1585), n (%)	The Czech Republic (n=1029), n (%)		France (n=385), n (%)		The Netherlands (n=2146), n (%)		Germany (n=4726), n (%)
**Sex**										
	Male	4725 (47.87)	824 (51.99)	347 (33.72)		167 (43.38)		1076 (50.14)		2311 (48.90)
	Female	5146 (52.13)	761 (48.01)	682 (66.28)^a^		218 (56.62)		1070 (49.86)		2415 (51.10)
**Age at the time of survey (years)**										
	18 to <25	1636 (16.57)	725 (45.74)	427 (41.50)		131 (34.03)		353 (16.45)		0 (0)
	25 to <30	2997 (30.36)	374 (23.60)	275 (26.72)		123 (31.95)		346 (16.12)		1879 (39.76)
	30 to <35	2463 (24.95)	239 (15.08)	164 (15.94)		93 (23.90)		402 (18.73)		1566 (33.14)
	35 to <40	1437 (14.56)	146 (9.21)	104 (10.11)		33 (8.57)		363 (16.92)		791 (16.74)
	40 to <45	833 (8.44)	63 (3.97)	49 (4.76)		6 (1.56)		301 (14.03)		414 (8.76)
	45-69	505 (5.12)	38 (2.40)	10 (0.97)		0 (0)		381 (17.75)		76 (1.61)
**Age at cancer diagnosis (years)**										
	0 to <5	3201 (32.43)	424 (26.75)	289 (28.09)		173 (44.94)		936 (43.62)		1379 (29.18)
	5 to <10	2572 (26.06)	352 (22.21)	196 (19.05)		97 (25.19)		595 (27.73)		1332 (28.18)
	10 to <15	3448 (34.93)	540 (34.07)	336 (32.65)		115 (29.87)		468 (21.81)		1989 (42.09)
	15-18	650 (6.58)	269 (16.97)	208 (20.21)		0 (0)		147 (6.85)		26 (0.55)
**Period of cancer diagnosis**										
	1963 to <1985	2086 (21.13)	340 (21.45)	53 (5.15)		0 (0)		711 (33.13)		982 (20.78)
	1985 to <1995	4680 (47.41)	721 (45.49)	283 (27.50)		261 (67.79)		764 (35.60)		2651 (56.09)
	1995 to <2005	2792 (28.28)	403 (25.43)	501 (48.69)		124 (32.21)		671 (31.27)		1093 (23.13)
	2005-2010	313 (3.17)	121 (7.63)	192 (18.66)		0 (0)		0 (0)		0 (0)
**Time since cancer diagnosis (years)**										
	5 to <10	455 (4.61)	291 (18.36)	164 (15.94)		0 (0)		0 (0)		0 (0)
	10 to <15	835 (8.46)	281 (17.73)	226 (21.96)		4 (1.04)		0 (0)		324 (6.86)
	15 to <20	2179 (22.07)	397 (25.05)	283 (27.50)		134 (34.81)		450 (20.97)		915 (19.36)
	20 to <25	2503 (25.36)	332 (20.95)	160 (15.55)		209 (54.29)		439 (20.46)		1363 (28.84)
	25 to <30	2023 (20.49)	183 (11.55)	130 (12.63)		38 (9.87)		393 (18.31)		1279 (27.06)
	30 to <35	1302 (13.19)	84 (5.30)	43 (4.18)		0 (0)		331 (15.42)		844 (17.86)
	35-54	574 (5.82)	17 (1.07)	23 (2.24)		0 (0)		533 (24.84)		1 (0.02)
**Cancer diagnosis (ICCC-3)^b^**										
	I Leukemias	3157 (31.97)	492 (31.04)	267 (25.95)		0 (0)^c^		724 (33.74)		1673 (35.40)
	II Lymphomas	2075 (21.02)	351 (22.15)	266 (25.85)		83 (21.56)		349 (16.26)		1026 (21.71)
	III CNS^d^ tumors	1356 (13.74)	236 (14.89)	125 (12.15)		96 (24.94)		256 (11.93)		643 (13.61)
	IV Neuroblastoma	440 (4.47)	51 (3.22)	46 (4.47)		52 (13.51)		118 (5.50)		174 (3.68)
	V Retinoblastoma	172 (1.74)	32 (2.02)	19 (1.85)		9 (2.34)		10 (0.47)		103 (2.18)
	VI Renal tumors	738 (7.48)	80 (5.05)	81 (7.87)		52 (13.51)		239 (11.14)		286 (6.05)
	VII Hepatic tumors	61 (0.62)	9 (0.57)	11 (1.07)		9 (2.34)		16 (0.75)		16 (0.34)
	VIII Bone tumors	588 (5.96)	94 (5.93)	62 (6.03)		30 (7.79)		116 (5.41)		286 (6.05)
	IX Soft tissue sarcomas	654 (6.63)	99 (6.25)	55 (5.34)		26 (6.75)		164 (7.64)		309 (6.54)
	X Germ cell tumors	386 (3.91)	60 (3.79)	60 (5.83)		15 (3.90)		88 (4.10)		163 (3.45)
	XI Epithelial neoplasms and melanomas	130 (1.32)	23 (1.45)	20 (1.94)		13 (3.38)		30 (1.40)		44 (0.93)
	Other malignant neoplasms^e^	114 (1.15)	58 (3.66)	17 (1.65)		0 (0)		36 (1.68)		3 (0.06)
**Subsequent tumor^f^**										
	Yes	646 (6.54)	45 (2.84)	64 (6.22)		14 (3.64)		149 (6.94)		374 (7.91)
	No	9225 (93.46)	1540 (97.16)	965 (93.78)		371 (96.36)		1997 (93.06)		4352 (92.09)
**Cancer treatment**										
	Surgery only	642 (6.50)	190 (11.99)	66 (6.41)		92 (23.90)		213 (9.93)		81(1.71)
	Radiotherapy only	49 (0.50)	2 (0.13)	2 (0.19)		2 (0.52)		26 (1.21)		17(0.36)
	Chemotherapy only	2086 (21.13)	354 (22.33)	176 (17.10)		35 (9.09)		639 (29.78)		882 (18.66)
	Surgery and radiotherapy	285 (2.89)	77 (4.86)	32 (3.11)		13 (3.38)		113 (5.27)		50 (1.06)
	Surgery and chemotherapy	1737 (17.60)	348 (21.96)	214 (20.80)		129 (33.51)		529 (24.65)		517 (10.94)
	Radiotherapy and chemotherapy	1975 (20.01)	145 (9.15)	249 (24.20)		30 (7.79)		257 (11.98)		1294 (27.38)
	Radiotherapy, chemotherapy, and surgery	1847 (18.71)	367 (23.15)	261 (25.36)		79 (20.52)		343 (15.98)		797 (16.86)
	No surgery, chemotherapy, or radiotherapy	46 (0.47)	16 (1.01)	3 (0.29)		3 (0.78)		18 (0.84)		6 (0.13)
	Complete treatment information not available	1204 (12.20)	86 (5.43)	26 (2.53)		2 (0.52)		8 (0.37)		1082 (22.89)
**Hematopoietic stem cell transplantation**										
	Unknown	307 (3.11)	51 (3.22)	3 (0.29)		0 (0)		4 (0.19)		249 (5.27)
	Yes	307 (3.11)	65 (4.10)	120 (11.66)		25 (6.49)		50 (2.33)		47 (0.99)
	No	9257 (93.78)	1469 (92.68)	906 (88.05)		360 (93.51)		2092 (97.48)		4430 (93.74)

^a^The Czech cohort included a higher proportion of women than other countries, because women were prioritized during recruitment as they were also part of an associated PanCareLIFE study on female fertility.

^b^ICCC-3: International Classification of Childhood Cancer, 3rd edition.

^c^Percentages of French cohort varied from those of other countries because the French cohort did not include survivors of leukemia and CNS tumors represented the largest of diagnostic groups.

^d^CNS: central nervous system.

^e^ICCC-3 main group XII (Other and unspecified malignant neoplasms) and Langerhans cell histiocytosis but not benign and in situ tumors and tumor-like lesions or unclassified survivors.

^f^All subsequent tumors registered in national registry until the start of the study.

Most survivors were diagnosed as having cancer between the ages of 10 and 14 years (3488/9871, 34.93%) or <5 years (3201/9871, 32.43%). Almost half (4680/9871, 47.41%) were diagnosed as having cancer between 1985 and 1994, most were survivors of leukemia (3157/9871, 31.97%), lymphoma (2075/9871, 21.02%), or CNS tumors (1356/9871, 13.74%), had no history of a subsequent tumor (9225/9871, 93.46%), and received chemotherapy with or without surgery and/or radiotherapy (7645/9871, 77.45%). More than half (5460/9871, 55.31%) of the participants were aged 25 to 34 years at the time of the HRQoL study.

Response rates differed between countries. The proportions of eligible survivors who were invited to participate were 83.13% (2513/3023) in Switzerland and 82.99% (10,078/12,144) in Germany, 80.28% (861/1060) in France, 75.37% (4250/5639) in the Netherlands, and 50.40% (1576/3127) in the Czech Republic (Table 3). The proportion of responders was highest in the Czech Republic, with 65.3% (1029/3127) and lowest in France (385/1060, 45.2%).

Participants were more often women, slightly younger at the time of the survey, and slightly older at cancer diagnosis; their cancer diagnosis tended to be in more recent years, and they were more often survivors of leukemia or lymphoma and less often survivors of CNS tumors than nonparticipants (Table 6). The Czech participants had the highest proportion of women (682/3127, 66.3%) because women were prioritized during recruitment as they were also part of an associated PanCareLIFE study on female fertility [25]. In other countries, the proportion of women ranged between 48.0% (761/3023, Switzerland) and 56.6% (218/1060, France).

**Table 6:** Comparison of participants and nonparticipants.

Characteristics		Participants (n=9871), n (%)	Nonparticipants (n=15,179), n (%)		P value^a^
**Sex**				<.001	
	Male	4725 (47.87)	9058 (59.67)		
	Female	5146 (52.13)	6121 (40.33)		
**Age at the time of survey (years)**				<.001	
	18 to <25	1636 (16.57)	2245 (14.79)		
	25 to <30	2997 (30.36)	4355 (28.69)		
	30 to <35	2463 (24.95)	3886 (25.60)		
	35 to <40	1437 (14.56)	2496 (16.44)		
	40 to <45	833 (8.44)	1399 (9.22)		
	45-69	505 (5.12)	798 (5.26)		
**Age at the time of cancer diagnosis (years)**				.005	
	0 to <5	3201 (32.43)	5075 (33.43)		
	5 to <10	2572 (26.06)	4055 (26.71)		
	10 to <15	3448 (34.93)	4977 (32.79)		
	15-18	650 (6.58)	1072 (7.06)		
**Period of cancer diagnosis**				.004	
	1963 to <1985	2086 (21.13)	3500 (23.06)		
	1985 to <1995	4680 (47.41)	7064 (46.54)		
	1995 to <2005	2792 (28.28)	4128 (27.20)		
	2005-2010	313 (3.17)	487 (3.21)		
**Time since cancer diagnosis (years)**				<.001	
	5 to <10	455 (4.61)	679 (4.47)		
	10 to <15	835 (8.46)	1024 (6.75)		
	15 to <20	2179 (22.07)	2986 (19.67)		
	20 to <25	2503 (25.36)	3738 (24.63)		
	25 to <30	2023 (20.49)	3513 (23.14)		
	30 to <35	1302 (13.19)	2335 (15.38)		
	35-54	574 (5.82)	904 (6.96)		
**Cancer diagnosis (ICCC-3)^b^**				<.001	
	I Leukemias	3157 (31.97)	4398 (28.97)		
	II Lymphomas	2075 (21.02)	3015 (19.86)		
	III CNS^c^ tumors	1356 (13.74)	2659 (17.52)		
	IV Neuroblastoma	440 (4.47)	696 (4.59)		
	V Retinoblastoma	172 (1.74)	320 (2.11)		
	VI Renal tumors	738 (7.48)	983 (6.48)		
	VII Hepatic tumors	61 (0.62)	101 (0.67)		
	VIII Bone tumors	588 (5.96)	983 (6.48)		
	IX Soft tissue sarcomas	654 (6.63)	954 (6.28)		
	X Germ cell tumors	386 (3.91)	644 (4.24)		
	XI Epithelial neoplasms and melanomas	130 (1.32)	279 (1.84)		
	Other malignant neoplasms^d^	114 (1.15)	147 (0.97)		
**Treatment**				<.001	
	Surgery only	642 (6.50)	1240 (8.17)		
	Radiotherapy only	49 (0.50)	87 (0.57)		
	Chemotherapy only	2086 (21.13)	2551 (16.81)		
	Surgery and radiotherapy	285 (2.89)	431 (2.84)		
	Surgery and chemotherapy	1737 (17.60)	2048 (13.49)		
	Radiotherapy and chemotherapy	1975 (20.01)	2580 (17.00)		
	Radiotherapy, chemotherapy, and surgery	1847 (18.71)	2075 (13.67)		
	No surgery, chemotherapy, or radiotherapy	46 (0.47)	76 (0.50)		
	Complete treatment information not available	1204 (12.20)	4091 (26.95)		

^a^P values were derived from Pearson chi-square tests.

^b^ICCC-3: International Classification of Childhood Cancer, 3rd edition.

^c^CNS: central nervous system.

^d^ICCC-3 main group XII (other and unspecified malignant neoplasms) and Langerhans cell histiocytosis but not benign and in situ tumors and tumor-like lesions or unclassified survivors.

## Discussion

### Central Message

PanCareLIFE has successfully constructed a large population of 9871 childhood cancer survivors with comprehensive data to assess HRQoL and its predictors. This rich data set of survivor cohorts from 5 different countries will allow in-depth investigation of the differences in HRQoL between countries, including the effect of female fertility and hearing impairment on HRQoL. The sample size and the wide range of treatment era, type of diagnosis, and age at diagnosis provide an excellent basis for risk stratification. It will thereby provide new scientific information on risk factors for impaired HRQoL after childhood cancer. This study will be the first to estimate the association between well-defined female fertility information and HRQoL with ample statistical power. Our results will help caregivers to identify survivors at risk for decreased HRQoL and will contribute to the development of evidence-based interventions toward better HRQoL of future childhood cancer survivors. Our data allow us to investigate similar predictors of HRQoL as in previous studies [6,11] (except for annual income) and, in addition, the role of female fertility impairment.

### Strengths and Limitations

This study is one of the largest studies worldwide to examine HRQoL and its predictors in childhood cancer survivors. It is, in terms of size, comparable with large studies from the United States (CCSS) [12] and the United Kingdom (BCCSS) [11]. This study combines a series of population-based or regionally well-defined cohorts from different European countries or regions. This will allow comparisons between national cohorts and representative in-depth analyses of HRQoL in survivors and their influencing factors. The central data center processed the raw data from all institutions to minimize coding and data cleaning errors before data pooling.

We faced some challenges when assembling the data from 6 institutions from 5 countries, as recruitment and study design differed between countries. Some countries had specific exclusion criteria before they invited the survivors to the survey, which may have led to selection biases. The Netherlands, the Czech Republic, and France excluded survivors with severe mental sequelae and other severe impairments. However, these were only a few patients (only 0.1% of eligible patients, n=28), and therefore, a potential bias toward an overestimation of HRQoL in these countries is negligible. Germany did not contact survivors <25 years on the date of the survey, France did not include survivors of childhood leukemia, and the Netherlands did not send data on hearing impairment. Depending on the research question, we will stratify the overall data set by age at survey (18-24 years and ≥25 years) and/or by country (including or excluding Germany and/or France and/or the Netherlands). The time elapsed between diagnosis and survey differed between countries. In Switzerland and the Czech Republic, survivors received a questionnaire already ≥5 years after diagnosis, whereas in Germany, France, and the Netherlands, it was much longer (≥10 years). However, in the SCCSS, the time since diagnosis did not influence HRQoL after adjusting for age at the time of the study [6]. We will, therefore, include age at the time of diagnosis and age at the time of the survey in all multivariable analyses. The Motol Teaching Hospital in Prague preferred female survivors to participate in the study. A high proportion of women may lead to the underestimation of HRQoL because women reported lower HRQoL than men in both CCSS [12] and BCCSS [11]. We will adjust for sex in all regression models and stratify the results by sex.

Of all countries, France had the highest percentage of survivors who were lost to follow-up and the Netherlands had the lowest. In the Netherlands, the proportion of 5-year survivors who died before the mailing of the questionnaire was highest, most probably because the Dutch cohort had the longest follow-up. Age at the time of study was a predictor for some domains of HRQoL in the SCCSS [6] and in a study from the European Organization for Research and Treatment of Cancer [28] pooling data from the general population. In future analyses, we will adjust for age at the time of the survey in multivariable regression models.

Different assessment logistics among the countries may have influenced response rates. In the Czech cohort, where most survivors were asked to participate during clinical follow-up visits, the participation rate was higher than in Switzerland, the Netherlands, or Germany, who invited all eligible participants via letters. Survivors who have a high risk for late effects are followed up with clinical visits more often than those with lower risk [29]. In the SCCSS, survivors with late effects were at risk for low HRQoL [6]. Therefore, the different sampling procedures in the Czech cohort could have resulted in a lower HRQoL score compared with the other countries. Differences in mentality and general willingness to take part in surveys may also explain the differences in response rates.

The participating survivors differed only slightly from those who did not respond, suggesting that bias from selective response may not be large. The higher proportion of participating women than men reflects a commonly observed self-selection bias in questionnaire health surveys [30]. The frequencies of observed diagnostic groups are similar to those reported from population-based cancer registries, with the following exceptions: more survivors of lymphoma and less survivors of brain tumors participated, reflecting a prognosis-based disease bias.

The countries contributing to this study assessed treatment data differently: most countries collected *as-treated* data retrospectively from medical records. Switzerland complemented intention-to-treat (ITT) data, with retrospective data from medical records. Germany used solely ITT data from a study protocol database, which did not have treatment data information for 23% of the survivors.

Overall, the limitations refer to different aspects of the data and are mostly specific for each country; therefore, we will provide overall and country-specific results and perform sensitivity analyses excluding countries with specific properties. We will also include a careful evaluation of the differences in data ascertainment and data quality between countries when interpreting the results for specific research questions.

### Conclusions and Prospects for Further Research

With careful interpretation, the large data set of this PanCareLIFE study provides a unique opportunity to study long-term HRQoL among childhood cancer survivors across Europe. It will contribute to the knowledge on HRQoL after childhood cancer while acknowledging the differences between countries in treatment traditions and long-term care. It will also allow the investigation of the role of female fertility and hearing impairment on HRQoL. The results may uncover unknown risk factors for reduced HRQoL and will help inform clinicians about certain groups of survivors who have greater needs for counseling and psychological support to obtain the best possible HRQoL.

## Appendix

Multimedia Appendix 1 Overview of outcome variables, exposures, and confounders for the regression analyses.

Multimedia Appendix 2 Flow diagram of the study sample, from eligible survivors to those included in the health-related quality of life (HRQoL) analyses, and planned subsamples.

## Abbreviations

AT: as treated
BCCSS: British Childhood Cancer Survivor Study
CCSS: Childhood Cancer Survivor Study
CNS: central nervous system
DCOG LATER: Dutch Childhood Oncology Group Survivor Study
FNM: Motol Teaching Hospital, Prague, the Czech Republic
FP7: European 7th Framework Program
GCCR: German Childhood Cancer Registry
HRQoL: health-related quality of life
ICCC-3: International Classification of Childhood Cancer, 3rd edition
ITT: intention to treat
SCCR: Swiss Childhood Cancer Registry
SCCSS: Swiss Childhood Cancer Survivor Study
SF-36: Short Form 36 Health Survey
VIVE: First Basic Survey on Life Situation, State of Health, and Quality of Life of Childhood Cancer Survivors in Germany

## References

[1] Gatta G, Botta L, Rossi S, Aareleid T, Bielska-Lasota M, Clavel J, Dimitrova N, Jakab Z, Kaatsch P, Lacour B, Mallone S, Marcos-Gragera R, Minicozzi P, Sánchez-Pérez MJ, Sant M, Santaquilani M, Stiller C, Tavilla A, Trama A, Visser O, Peris-Bonet R, EUROCARE Working Group (2014). Childhood cancer survival in Europe 1999-2007: results of EUROCARE-5--a population-based study. Lancet Oncol.

[2] Vassal G, Schrappe M, Pritchard-Jones K, Arnold F, Basset L, Biondi A, Bode G, Eggert A, Hjorth L, Kamerić L, Kamerić N, Karner S, Kearns P, Kienesberger A, Kowalczyk J, Lack P, Perilongo G, Sullivan R, Tsirou A, Essiaf S, Ladenstein R (2016). The SIOPE strategic plan: a European cancer plan for children and adolescents. J Cancer Policy.

[3] Hudson MM, Ness KK, Gurney JG, Mulrooney DA, Chemaitilly W, Krull KR, Green DM, Armstrong GT, Nottage KA, Jones KE, Sklar CA, Srivastava DK, Robison LL (2013). Clinical ascertainment of health outcomes among adults treated for childhood cancer. J Am Med Assoc.

[4] Friend AJ, Feltbower RG, Hughes EJ, Dye KP, Glaser AW (2018). Mental health of long-term survivors of childhood and young adult cancer: a systematic review. Int J Cancer.

[5] Bhakta N, Liu Q, Ness KK, Baassiri M, Eissa H, Yeo F, Chemaitilly W, Ehrhardt MJ, Bass J, Bishop MW, Shelton K, Lu L, Huang S, Li Z, Caron E, Lanctot J, Howell C, Folse T, Joshi V, Green DM, Mulrooney DA, Armstrong GT, Krull KR, Brinkman TM, Khan RB, Srivastava DK, Hudson MM, Yasui Y, Robison LL (2017). The cumulative burden of surviving childhood cancer: an initial report from the St Jude lifetime cohort study (SJLIFE). Lancet.

[6] Rueegg CS, Gianinazzi ME, Rischewski J, Popovic MB, von der Weid NX, Michel G, Kuehni CE (2013). Health-related quality of life in survivors of childhood cancer: the role of chronic health problems. J Cancer Surviv.

[7] Langeveld NE, Grootenhuis MA, Voûte PA, de Haan RJ, van den Bos C (2004). Quality of life, self-esteem and worries in young adult survivors of childhood cancer. Psychooncology.

[8] Thouvenin-Doulet S, Berger C, Casagranda L, Oberlin O, Marec-Berard P, Pacquement H, Guibout C, Freycon C, N'Guyen TD, Bondiau P, Laprie A, Berchery D, El-Fayech C, Trombert-Paviot B, de Vathaire F (2018). Fecundity and quality of life of women treated for solid childhood tumors between 1948 and 1992 in France. J Adolesc Young Adult Oncol.

[9] de Haan R, Aaronson N, Limburg M, Hewer RL, van Crevel H (1993). Measuring quality of life in stroke. Stroke.

[10] Lund LW, Schmiegelow K, Rechnitzer C, Johansen C (2011). A systematic review of studies on psychosocial late effects of childhood cancer: structures of society and methodological pitfalls may challenge the conclusions. Pediatr Blood Cancer.

[11] Reulen RC, Winter DL, Lancashire ER, Zeegers MP, Jenney ME, Walters SJ, Jenkinson C, Hawkins MM (2007). Health-status of adult survivors of childhood cancer: a large-scale population-based study from the British childhood cancer survivor study. Int J Cancer.

[12] Hudson MM, Mertens AC, Yasui Y, Hobbie W, Chen H, Gurney JG, Yeazel M, Recklitis CJ, Marina N, Robison LR, Oeffinger KC, Childhood Cancer Survivor Study Investigators (2003). Health status of adult long-term survivors of childhood cancer: a report from the childhood cancer survivor study. J Am Med Assoc.

[13] Byrne J, Grabow D, Campbell H, O'Brien K, Bielack S, Zehnhoff-Dinnesen AA, Calaminus G, Kremer L, Langer T, van den Heuvel-Eibrink MM, van Dulmen-den Broeder E, Baust K, Bautz A, Beck JD, Berger C, Binder H, Borgmann-Staudt A, Broer L, Cario H, Casagranda L, Clemens E, Deuster D, de Vries A, Dirksen U, Winther JF, Fosså S, Font-Gonzalez A, Grandage V, Haupt R, Hecker-Nolting S, Hjorth L, Kaiser M, Kenborg L, Kepak T, Kepáková K, Knudsen LE, Krawczuk-Rybak M, Kruseova J, Kuehni CE, Kunstreich M, Kuonen R, Lackner H, Leiper A, Loeffen EA, Luks A, Modan-Moses D, Mulder R, Parfitt R, Paul NW, Ranft A, Ruud E, Schilling R, Spix C, Stefanowicz J, Strauβ G, Uitterlinden AG, van den Berg M, van der Kooi A, van Dijk M, van Leeuwen F, Zolk O, Zöller D, Kaatsch P, PanCareLIFE consortium (2018). PanCareLIFE: the scientific basis for a European project to improve long-term care regarding fertility, ototoxicity and health-related quality of life after cancer occurring among children and adolescents. Eur J Cancer.

[14] The PanCareLIFE Project. PanCareLIFE – Life after Childhood Cancer.

[15] Kuehni CE, Rueegg CS, Michel G, Rebholz CE, Strippoli MP, Niggli FK, Egger M, von der Weid NX, Swiss Paediatric Oncology Group (SPOG) (2012). Cohort profile: the Swiss childhood cancer survivor study. Int J Epidemiol.

[16] Schindler M, Mitter V, Bergstraesser E, Gumy-Pause F, Michel G, Kuehni CE, Swiss Paediatric Oncology Group (SPOG) (2015). Death certificate notifications in the Swiss childhood cancer registry: assessing completeness and registration procedures. Swiss Med Wkly.

[17] Teepen JC, van Leeuwen FE, Tissing WJ, van Dulmen-den Broeder E, van den Heuvel-Eibrink MM, van der Pal HJ, Loonen JJ, Bresters D, Versluys B, Neggers SJCMM, Jaspers MWM, Hauptmann M, van der Heiden-van der Loo M, Visser O, Kremer LCM, Ronckers CM, DCOG LATER Study Group (2017). Long-Term Risk of Subsequent Malignant Neoplasms After Treatment of Childhood Cancer in the DCOG LATER Study Cohort: Role of Chemotherapy. J Clin Oncol.

[18] Winther JF, Kenborg L, Byrne J, Hjorth L, Kaatsch P, Kremer LC, Kuehni CE, Auquier P, Michel G, de Vathaire F, Haupt R, Skinner R, Madanat-Harjuoja LM, Tryggvadottir L, Wesenberg F, Reulen RC, Grabow D, Ronckers CM, van Dulmen-den Broeder E, van den Heuvel-Eibrink MM, Schindler M, Berbis J, Holmqvist AS, Gudmundsdottir T, de Fine SL, Bonnesen TG, Asdahl PH, Bautz A, Kristoffersen AK, Himmerslev L, Hasle H, Olsen JH, Hawkins MM (2015). Childhood cancer survivor cohorts in Europe. Acta Oncol.

[19] Grabow D, Spix C, Blettner M, Kaatsch P (2011). Strategy for long-term surveillance at the German childhood cancer registry - an update. Klin Padiatr.

[20] Kaatsch P, Grabow D (2012). The German cohort of long-term survivors of childhood cancer. A population-based cohort in the German childhood cancer registry. Bundesgesundheitsblatt Gesundheitsforschung Gesundheitsschutz.

[21] Steliarova-Foucher E, Stiller C, Lacour B, Kaatsch P (2005). International classification of childhood cancer, third edition. Cancer.

[22] Ware JE, Gandek B (1998). Overview of the SF-36 health survey and the international quality of life assessment (IQOLA) project. J Clin Epidemiol.

[23] Ware JE, Kosinski M, Dewey J (2001). How to Score Version 2 of the SF-36 Health Survey.

[24] Ellert U, Kurth BM (2013). Health related quality of life in adults in Germany: results of the German health interview and examination survey for adults (DEGS1). Bundesgesundheitsblatt Gesundheitsforschung Gesundheitsschutz.

[25] van den Berg M, van Dijk M, Byrne J, Campbell H, Berger C, Borgmann-Staudt A, Calaminus G, Dirksen U, Winther JF, Fossa SD, Grabow D, Grandage VL, van den Heuvel-Eibrink MM, Kaiser M, Kepak T, Kremer LC, Kruseova J, Kuehni CE, Lambalk CB, van Leeuwen FE, Leiper A, Modan-Moses D, Morsellino V, Spix C, Kaatsch P, van Dulmen-den Broeder E, PanCareLIFE Consortium (2018). Fertility among female survivors of childhood, adolescent, and young adult cancer: protocol for two pan-european studies (PanCareLIFE). JMIR Res Protoc.

[26] Ellert U, Bellach BM (1999). The SF-36 in the federal health survey-description of a current normal sample. Gesundheitswesen.

[27] Roser K, Mader L, Baenziger J, Sommer G, Kuehni CE, Michel G (2019). Health-related quality of life in Switzerland: normative data for the SF-36v2 questionnaire. Qual Life Res.

[28] Nolte S, Liegl G, Petersen MA, Aaronson NK, Costantini A, Fayers PM, Groenvold M, Holzner B, Johnson CD, Kemmler G, Tomaszewski KA, Waldmann A, Young TE, Rose M, EORTC Quality of Life Group (2019). General population normative data for the EORTC QLQ-C30 health-related quality of life questionnaire based on 15,386 persons across 13 European countries, Canada and the Unites States. Eur J Cancer.

[29] Gebauer J, Baust K, Bardi E, Grabow D, Stein A, van der Pal HJ, Calaminus G, Langer T (2020). Guidelines for long-term follow-up after childhood cancer: practical implications for the daily work. Oncol Res Treat.

[30] Koen B, Loosveldt G, Vandenplas C, Stoop I (2018). Response Rates in the European Social Survey: Increasing, Decreasing, or a Matter of Fieldwork Efforts? Survey Methods: Insights from the Field. Survey Methods: Insights from the Field.

