# Supplementary material for: Health-Related Quality of Life in European Childhood Cancer Survivors: Protocol for a Study Within PanCareLIFE
Source: JMIR Res Protoc. 2021 Jan 25;10(1):e21851. doi: 10.2196/21851 (PMC7870350; doi:10.2196/21851)
Supplement: Multimedia Appendix 1 [file resprot_v10i1e21851_app1.pdf]

Multimedia Appendix 1: Overview of outcome variables, exposures, and confounders for the regression analyses

Variable	(1) Predictors of HRQoL	(2) Effect of fertility impairment on HRQoL	(3) Effect of hearing impairment on HRQoL
Country[a]	E	E	E
**Demographic variables**			
Gender[a]	E	C	C
Age at survey[b]	E	C	C
**Socio-economic variables**			
Migration background[b]	E	C	C
Education[b]	E	E	E
Occupational status[b]	E	E	E
Living with a partner[b]	E	C	E
**Lifestyle variables**			
Smoking status[b]	E	C	E
Alcohol consumption[b]	E	C	E
Body Mass Index[b]	E	C	E
**Cancer-related variables**			
Age at cancer diagnosis[a]	E	C	C
Time since cancer diagnosis[a]	E	C	C
Type of cancer diagnosis[a]	E	C	C
Relapse[a]	E	C	C
**Treatment-related variables**			
Surgery[a]	E	C	C
Radiotherapy[a]	E	C	C
Chemotherapy[a]	E	C	C
Bone marrow transplantation[a]	E	C	C
**Fertility-related variables**			
Fertility impairment[c]		E	
**Hearing-related variables**			
Hearing impairment[b]			E
**HRQoL variables**			
Physical Functioning[b]		O	O
Bodily Pain[b]		O	O
Role Limitation Physical[b]		O	O
Energy & Vitality[b]		O	O
Mental Health[b]		O	O
General Health Perception[b]		O	O
Role Limitation Emotional[b]		O	O
Social Functioning[b]		O	O
Physical Component Summary[b]	O	O	O
Mental Component Summary[b]	O	O	O

Abbreviations: HRQoL, health-related quality of life; C, confounder; E, exposure; O, outcome variable.

[a] extracted from medical documents, [b] self-report, [c] from self-report and medical documents
