# Supplementary material for: Health-Related Quality of Life in European Childhood Cancer Survivors: Protocol for a Study Within PanCareLIFE
Source: JMIR Res Protoc. 2021 Jan 25;10(1):e21851. doi: 10.2196/21851 (PMC7870350; doi:10.2196/21851)
Supplement: Multimedia Appendix 2 [file resprot_v10i1e21851_app2.pdf]

Multimedia Appendix 2: Flow diagram of the study sample, from eligible survivors to those included in the HRQoL[a] analyses, and planned subsamples.

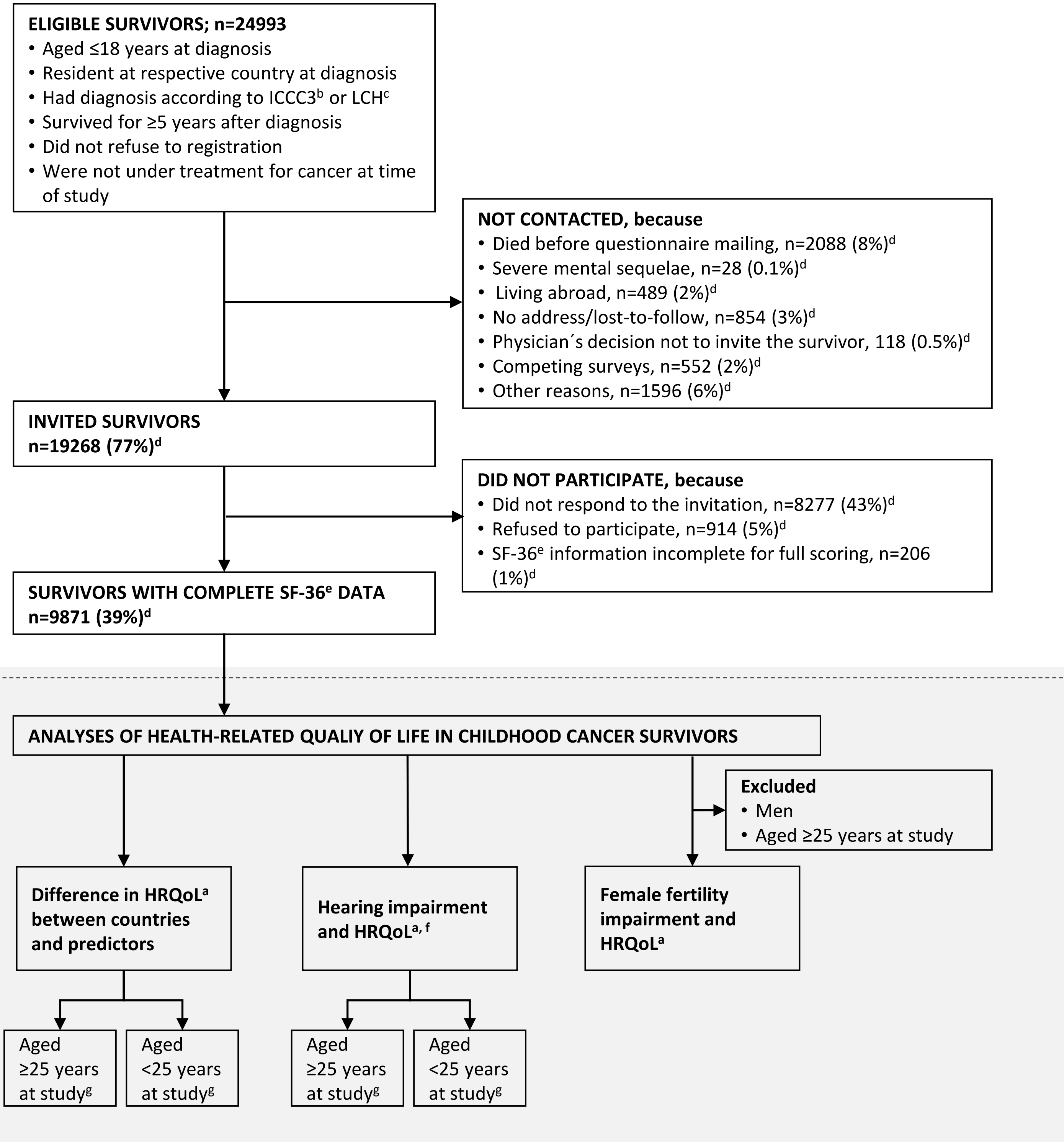

[a] HRQoL: health-related quality of life

[b] ICCC-3: International Classification of Childhood Cancer, 3rd edition.

[c] LCH: Langerhans cell histiocytosis

[d] Proportions based on eligible survivors.

[e] SF-36: Short Form-36

[f] Data on hearing problems were not available for the Netherlands.

[g]Because Germany excluded survivors survivors <25 years at time of study, we will run two main subanalyses: i) For survivors ≥25 years including survivors from all countries; and ii) For survivors <25 years (without Germany).
